# Identification of prognostic biomarkers in glioblastoma using a long non-coding RNA-mediated, competitive endogenous RNA network

**DOI:** 10.18632/oncotarget.9569

**Published:** 2016-05-24

**Authors:** Yuze Cao, Peng Wang, Shangwei Ning, Wenbiao Xiao, Bo Xiao, Xia Li

**Affiliations:** ^1^ Department of Neurology, Xiangya Hospital, Central South University, Changsha, 410008, Hunan Province, China; ^2^ College of Bioinformatics Science and Technology, Harbin Medical University, Harbin, 150081, Heilongjiang Province, China

**Keywords:** lncRNA, ceRNA network, topology, prognostic biomarker, synergistic regulation

## Abstract

Glioblastoma multiforme (GBM) is a highly malignant brain tumor associated with a poor prognosis. Cross-talk between competitive endogenous RNAs (ceRNAs) plays a critical role in tumor development and physiology. In this study, we present a multi-step computational approach to construct a functional GBM long non-coding RNA (lncRNA)-mediated ceRNA network (LMCN) by integrating genome-wide lncRNA and mRNA expression profiles, miRNA-target interactions, functional analyses, and clinical survival analyses. LncRNAs in the LMCN exhibited specific topological features consistent with a regulatory association with coding mRNAs across GBM pathology. We determined that the lncRNA MCM3AP-AS was involved in RNA processing and cell cycle-related functions, and was correlated with patient survival. MCM3AP-AS and MIR17HG acted synergistically to regulate mRNAs in a network module of the competitive LMCN. By integrating the expression profile of this module into a risk model, we stratified GBM patients in both the The Cancer Genome Atlas and an independent GBM dataset into distinct risk groups. Finally, survival analyses demonstrated that the lncRNAs and network module are potential prognostic biomarkers for GBM. Thus, ceRNAs could accelerate biomarker discovery and therapeutic development in GBM.

## INTRODUCTION

Glioblastoma multiforme (GBM) is an aggressive, malignant brain tumor [1]. There are approximately 10,000 new cases of high-grade glioma each year [2]. The prognosis is poor despite advances in treatment modalities (e.g., maximal surgical resection followed by irradiation and adjuvant temozolomide chemotherapy) that have improved survival [3]. While histological diagnosis based on morphology can provide valuable information regarding treatment, it is insufficient for predicting clinical outcomes [4]. There is an urgent need for suitable molecular biomarkers for GBM diagnosis and for predicting patient prognosis.

MicroRNAs (miRNAs) regulate gene expression at the post-transcriptional level and are involved in diverse biological processes and diseases [5]. Recently, long non-coding RNAs (lncRNAs) have received considerable attention [6]. These RNAs are non-protein-coding and are greater than 200 nucleotides in length. Owing to the development of high-throughput sequencing technology, lncRNAs have been discovered in a wide range of biological processes [7]. Recent studies have suggested that lncRNAs play complex and critical roles in tumor development and pathology [8]. For example, the expression of the oncogenic lncRNA MALAT1 was correlated with metastasis and patient survival in lung cancer [9] and GBM [8]. Additionally, the lncRNA H19 was shown to promote cancer development and invasion in glioma [10]. Finally, the lncRNA HOTAIR is highly expressed in metastatic breast cancer, and inhibition of HOTAIR expression may therefore prevent metastasis [11]. Over-expression of HOTAIR has been shown to promote epithelial ovarian cancer metastasis and was predictive of poor patient prognosis [12].

The functions of lncRNAs in GBM are not well characterized, and the identification of lncRNA biomarkers is challenging. Typically, a ‘guilt by association’ strategy has been used to characterize lncRNA function [13–15]. Recent studies have demonstrated that lncRNAs compete with endogenous RNAs (ceRNAs) by acting as miRNA sponges to regulate expression level of other transcripts [16–19]. For example, the lncRNA HULC plays an important regulatory role in lung cancer by acting as an endogenous ceRNA [20]. In addition, the muscle-specific lncRNA linc-MD1 regulates the timing of muscle differentiation by sequestering miR-133 to modulate the expression of MAML1 and MEF2C [21]. A recent study identified a lncRNA-associated ceRNA network across 12 different cancers and identified prognostic lncRNAs using network analysis [19]. Another study proposed that the ceRNA interaction network of GBM could reveal canonical oncogenic pathways [22]. Several ceRNA-related databases have also been developed to facilitate interference of lncRNA function [15, 19, 23]. Collectively, this data underscores the importance of lncRNA interactions with ceRNAs, and indicates that integration of expression profiles and network analysis could enable identification of risk lncRNAs and the underlying tumor pathology.

In this study, we used a multi-step computational approach to construct a functional lncRNA-mediated ceRNA network (LMCN) involved in GBM. We utilized the high-throughput molecular profiles of 422 GBM samples collected from The Cancer Genome Atlas (TCGA) [24]. Our systematic analysis entailed integrating genome-wide lncRNA/mRNA expression profiles, comprehensive miRNA-target interactions, functional analyses, and clinical survival analyses. Based on network analysis, we found that lncRNAs exhibited specific topological features in the LMCN, consistent with a regulatory association with coding mRNAs across GBM pathology. The lncRNA MCM3AP-AS was involved in RNA processing and cell cycle-related functions, and was significantly associated with survival in GBM patients. Additionally, we found that MCM3AP-AS and the lncRNA MIR17HG had a synergistic regulation of mRNA expression. By exploring a competitive sub-network within the LMCN, we determined that MCM3AP-AS and MIR17HG were involved in a ceRNA regulatory module and synergistically competed with MATR3, XPO1, and ZCCHC14. By integrating the expression profile of this module into a risk model, we stratified GBM patients from both the TCGA and another independent GBM dataset (GSE7696) into different risk groups. These analyses demonstrated that cancer-associated lncRNA-mediated ceRNA networks could be used to accelerate the discovery of molecular biomarkers and GBM therapeutics.

## RESULTS

### Construction of the LMCN

**Figure 1:**
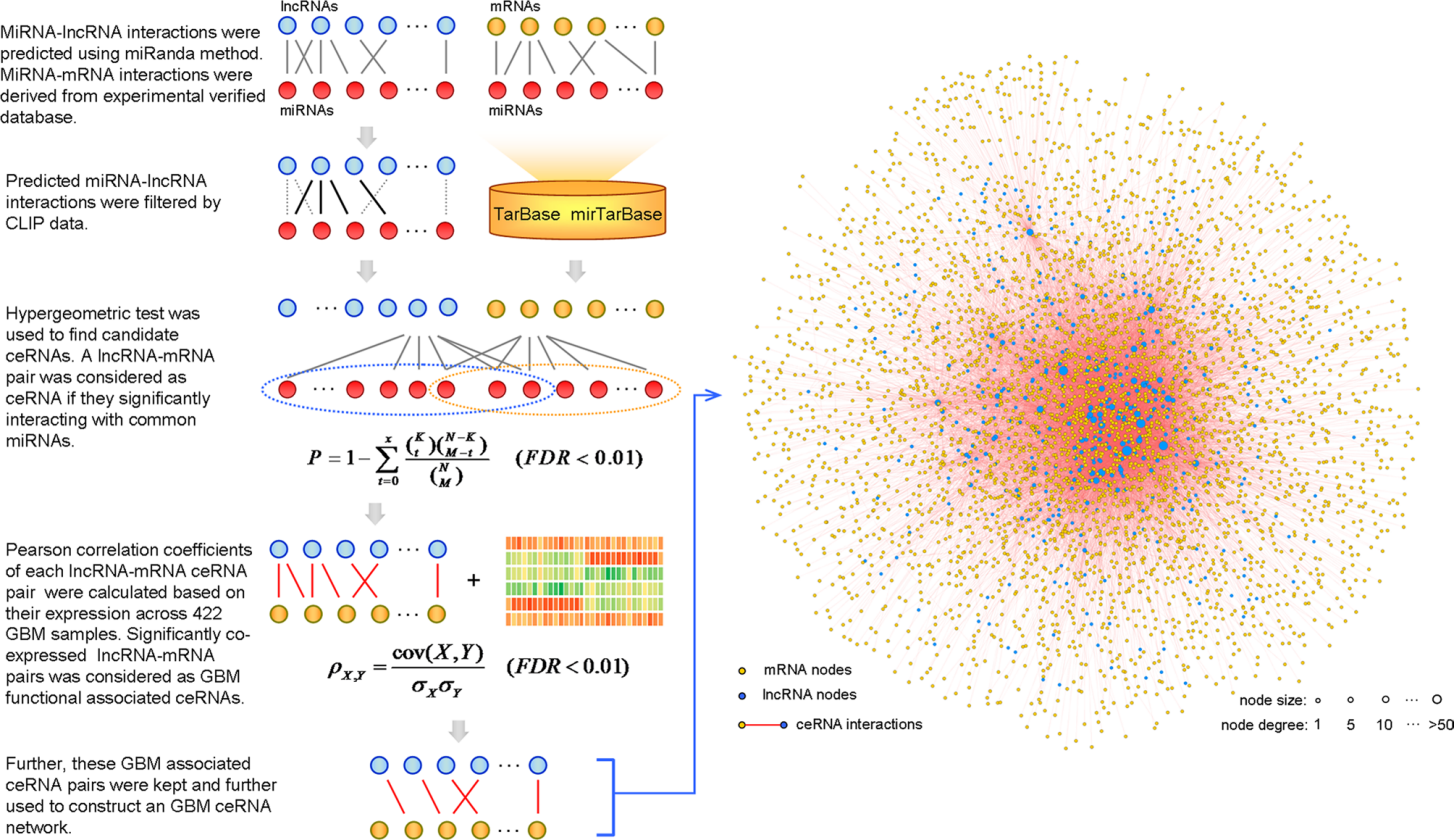
Construction of the LMCN and overview across 422 GBM patients The framework considered both computational prediction and experimental validation methods and datasets to identify functional lncRNA-mediated, competing interactions (left panel). Interactions between ceRNAs were illustrated and analyzed as a competing network.

To evaluate the lncRNA-mediated ceRNA interaction landscape, we constructed a LMCN using a multi-step approach. Previous studies have indicated that lncRNA transcripts compete with endogenous mRNAs to bind miRNAs [25], which can be identified using current miRNA target prediction methods [26–28]. The miRanda [29] method was first used to predict miRNA target sites in lncRNA transcripts (Figure 1). To identify experimentally verified miRNA-lncRNA interactions, we collected and integrated AGO-CLIP sequencing data into our method. For the miRNA-mRNA interactions, we used manually curated datasets from the TarBase and miRTarBase, which provide high confidence miRNA-target interactions supported by experiments. To identify candidate lncRNA-mRNA competing pairs, we used a hypergeometric test to compute the significance of shared common miRNAs between each lncRNA-mRNA pair. Significant pairs were retained for further identification. A number of ceRNAs are co-expressed in certain tissues and exhibit activities in specific cancers [19]. To identify active ceRNA pairs in GBM, we computed the Pearson correlation coefficient for each candidate ceRNA pair identified above. Finally, significantly co-expressed lncRNA-mRNA ceRNA pairs were used to construct the LMCN and were graphically modeled (Figure 1). The LMCN contained 393 lncRNAs, 4,176 mRNAs, and 16,860 ceRNA interactions.

### Biological and topological properties of the LMCN

**Figure 2:**
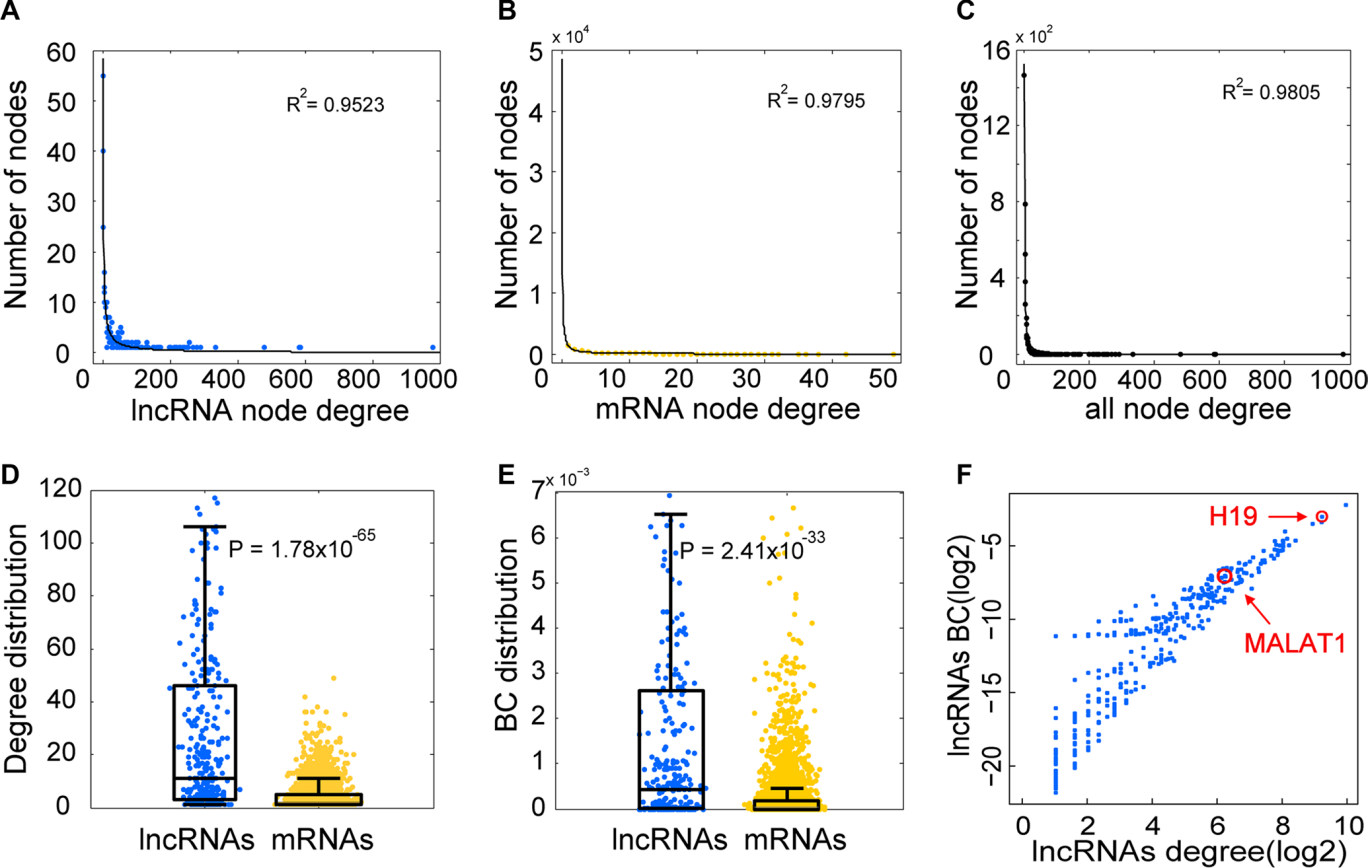
Topological analysis reveals specific properties of the LMCN The lncRNA nodes (**A**), mRNA nodes (**B**), and the entire network (**C**) reflect a power-law distribution. The node degree distribution of the lncRNAs was significantly higher than that of the mRNAs (**D**). The BC distribution of the lncRNAs was significantly higher than that of the mRNAs (**E**). Several well-known GBM associated lncRNAs were found in the hub and bottleneck nodes in the LMCN (**F**).

Based on the LMCN, we explored the structure and organization of the ceRNA interactions in GBM. The LMCN was separated into two layers using the Cytoscape software Organic layout. The lncRNA nodes were typically in the central region of the network, while the mRNAs nodes were typically in the outside layer (Figure 1). Crosstalk between ceRNAs can be modulated by multiple layers of gene regulation that involve interactions between diverse RNA species [30]. Based on ceRNA regulatory behavior, we concluded that the lncRNAs in the LMCN were more likely to have central regulatory roles than mRNAs. An investigation of the degree of the distribution of the lncRNA nodes (R^2^ = 0.95), the mRNA nodes (R^2^ = 0.98), and the entire network (R^2^ = 0.98) revealed power law distributions (Figure 2A–2C), which indicated that the GMB-associated LMCN was a scale-free network. These results suggested that the LMCN was similar to many biological networks and was well organized by a core set of lncRNA-mRNA competing principles into structured rather than random networks [31]. We considered two topological properties of the LMCN: the node degree and the betweenness centrality (BC). In general, a higher degree indicated that the node was a hub that participated in more ceRNA interactions. A higher BC implied that the node was a bottleneck that acted as bridges connecting different network modules. We found that lncRNA nodes usually had more degrees and BCs compared to mRNA nodes (Figure 2D, 2E). These observations indicated that although the lncRNAs did not code for proteins, they exhibited more specific topological properties than mRNAs in the LMCN.

We next analyzed the topological properties of well-known GBM-associated lncRNAs in the LMCN. In the lncRNADisease database [32], there were three lncRNAs associated with risk of GBM. Two of these lncRNAs, H19 and MALAT1, had annotated Ensembl IDs and were mapped to the LMCN. H19 was shown to have relatively high and low expression levels in CD133^+^ and CD133^−^ glioblastoma cells, respectively [33]. Additionally, knockdown of MALAT1 reduced GBM cell migration [34]. We found that both H19 and MALAT1 had a higher node degree and BC in the LMCN (Figure 2F), indicating that they were both hub and bottleneck nodes.

### Identification of lncRNAs associated with GBM prognosis

**Figure 3:**
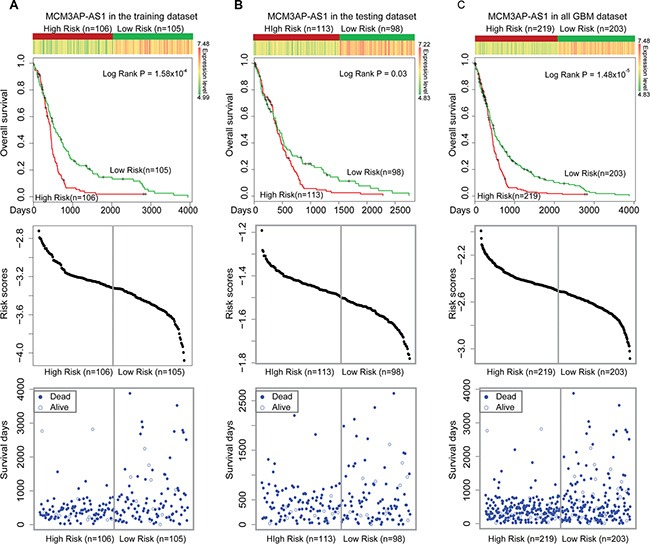
Survival analyses based on *MCM3AP-AS* expression (**A**) *MCM3AP-AS* expression could be used to divide the GBM patients in the training dataset into two different risk groups (log-rank test, *P* = 1.58 × 10^−4^). (**B**) *MCM3AP-AS* expression could be used to divide patients in the test group into two different risk groups (*P* = 0.03). (**C**) *MCM3AP-AS* expression could be used to divide all 422 GBM patients into two different risk groups (*P* = 1.48 × 10^−5^). The patients with high-risk scores were assigned into high-risk groups (associated with reduced survival times). The vertical gray lines represent the risk score thresholds in the survival analysis.

Based on the characteristics of the lncRNAs in the LMCN, we hypothesized that some hub and bottleneck nodes were risk lncRNAs for GBM. These properties have been utilized to infer which nodes were prognostic factors for cancer and to identify modules within the network [14, 35]. We performed a Cox regression analysis of the hub and bottleneck nodes to investigate whether lncRNAs with specific properties were prognostic factors in GBM. In previous studies, hubs were typically defined as the top 10–20% of the nodes in the networks [23, 36, 37]. We defined hubs as the top 5% (approximately the top 20 of 393 lncRNAs), which was a more stringent threshold. Similarly, bottleneck nodes were defined as the top 5% according to BC. Overall, 18 lncRNAs were defined as both hubs and bottleneck nodes. Five lncRNAs had a significant effect on survival (Table 1). To determine whether these lncRNAs were prognostic factors for GBM, we constructed a risk model (described in the Materials and Methods). We randomly assigned 422 GBM patients to two groups that were used as training (*n* = 211) and test (*n* = 211) datasets (Table 2). Kaplan-Meier survival analysis of the training dataset revealed that MCM3AP-AS could be used to divide the training GBM patients into two different risk groups (Figure 3A, *P* = 1.58 × 10^−4^). The high-risk group consisted of patients with high risk scores and had lower survival times (Figure 3A). Next, we investigated MCM3AP-AS in the test dataset using the same risk score threshold as that of the training set. The patients were divided into high- and low-risk groups (Figure 3B, *P* = 0.03). We used MCM3AP-AS as a prognostic biomarker in order to divide all 422 GBM patients into different groups (Figure 3C, *P* = 1.48 × 10^−5^). These results indicated that MCM3AP-AS was a protective factor for survival in GBM.

**Table 1:** Univariate Cox regression analysis of the hub and bottleneck lncRNAs in the LMCN

LncRNA	Ensembl ID	Univariate Cox analysis		
		HR (95% CI)	Coefficient	*P*-value
MCM3AP-AS	ENSG00000215424	0.6221 (0.5246–0.8356)	−0.4123	0.0005
NEAT1	ENSG00000245532	1.1703 (1.0432–1.3129)	0.1572	0.0073
LRRC75A-AS1	ENSG00000175061	0.7378 (0.5931–0.9177)	−0.3041	0.0063
RP11-175O19.4	ENSG00000231025	0.7928 (0.6591–0.9535)	−0.2322	0.0137
AC074117.10	ENSG00000234072	0.7661 (0.6229–0.9423)	−0.2663	0.0116

**Table 2:** Clinicopathologic characteristics of the TCGA GBM patients (*n* = 422)

Characteristic	Number of patients		
	All patients *n* = 422	Training set *n* = 211	Test set *n* = 211
Sex			
Male	262	129	133
Female	160	82	78
Age			
Mean ± SD	56.96 ± 14.73	56.00 ± 15.27	57.91 ± 14.14
Range	10–89	10–89	14–86
Histological type			
Treated primary GBM	17	12	5
Untreated primary GBM	405	199	206
History of neoadjuvant treatment			
Yes	20	12	8
No	402	199	203
Survival (months)			
Mean ± SD	18.24 ± 19.30	20.78 ± 22.38	15.70 ± 15.27
Range	0.10–129.37	0.20–129.37	0.10–88.27
State			
Alive	52	27	25
Dead	370	184	186

### MCM3AP-AS is involved in RNA processing and cell cycle-related functions

**Figure 4:**
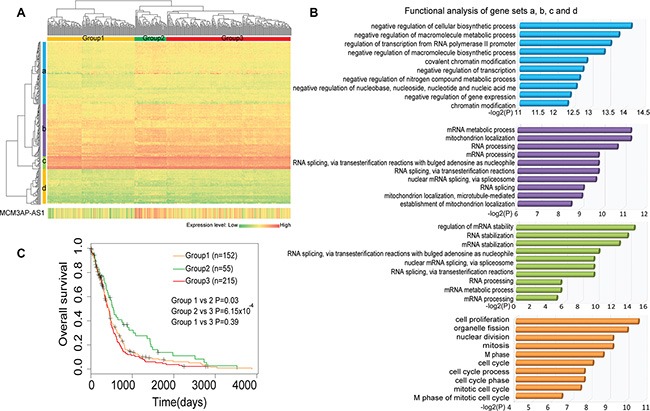
Comprehensive analysis of the expression and function of *MCM3AP-AS-* competing genes (**A**) A two-dimensional hierarchical analysis of the expression profiles of *MCM3AP-AS* and competing genes. The genes were clustered into four sets across three groups of patients. (**B**) Functional enrichment analysis of each clustered gene set reveals RNA processing and cell cycle-related functions. (**C**) A Kaplan-Meier survival analysis indicates group 2 patients had significantly higher survival times than group 1 (Figure 4C, log-rank test, *P* = 0.02) and group 3 (*P* = 6.15 × 10^−4^).

The hub and bottleneck properties of MCM3AP-AS indicated this lncRNA likely competed with other mRNAs and was connected to different components of the LMCN. According to the Ensembl Genome Browser, MCM3AP-AS is an antisense non-coding RNA that aligns with the coding-gene MCM3AP, which is one of the mini-chromosome maintenance proteins essential for the initiation of DNA replication (Supplementary Figure S1). To investigate the functions of MCM3AP-AS, we used a ‘guilt by association’ method [13, 19]. MCM3AP-AS interacted with 257 ceRNA neighbors in the LMCN. A hierarchical cluster analysis based on these 257 mRNAs revealed that 422 GBM patients could be divided into three groups (1, 2, and 3) with specific expression patterns (Figure 4A). MCM3AP-AS neighboring genes could generally be grouped into four different sets (a, b, c, and d). GBM Gene set functional enrichment analysis was then performed on each of the four gene sets based on Gene Ontology terms. The genes were significantly enriched in RNA processing and cell cycle-related functions (Figure 4B). Gene set a was associated with the negative regulation of RNA expression. Gene set b was associated with RNA splicing and mitochondrion localization, which is consistent with the function of MCM3AP. Gene set c was associated with RNA splicing, processing, and stabilization. Finally, gene set d was enriched in cell cycle processes such as M phase, nuclear division, and proliferation. We observed higher expression of MCM3AP-AS in group 2 compared to groups 1 and 3. Kaplan-Meier survival analysis indicated that the group 2 patients had significantly longer survival times than group 1 (Figure 4C; log-rank test, *P* = 0.02) and group 3 (*P* = 6.15 × 10^−4^).

### Identification of a highly competitive sub-network reveals prognostic ceRNA modules

**Figure 5:**
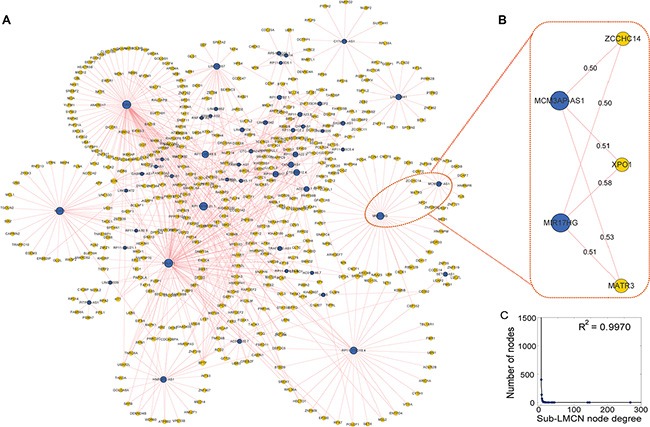
Overview of the highly competitive sub-network and module analysis (**A**) The sub-LMCN contains 52 lncRNA, 462 mRNA, and 653 ceRNA interactions. LncRNAs are shown as blue nodes and mRNAs are shown as yellow nodes. (**B**) Identification of a synergistic, co-competing module. Pearson correlation coefficients are provided for each edge. (**C**) The sub-LMCN had a scale-free structure with power law degree distributions.

While the LMCN could provide a global view of all possible competing ceRNA interactions that could be used to investigate the regulatory properties of the lncRNAs, the partial sub-networks revealed a more detailed picture of how the lncRNAs synergized with competing mRNAs. We derived a high-competing sub-network (sub-LMCN) from the LMCN by applying a Pearson correlation coefficient threshold > 0.5. This threshold was used in a previous study to identify functional activated (competing) ceRNA networks across 12 cancers [19]. The sub-LMCN contained 52 lncRNAs, 462 mRNAs, and 653 ceRNA interactions (Figure 5A). Similar to the LMCN, the sub-LMCN also had a scale-free structure with power law degree distributions (Figure 5C, R^2^ = 0.9970). GBM-associated lncRNAs such as MALAT1 [34] and MCM3AP-AS (identified in this study) competed with other mRNAs in the sub-LMCN.

**Figure 6:**
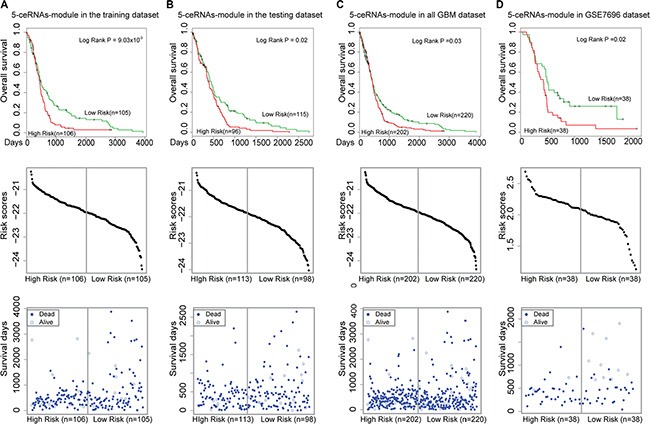
Survival analysis of the 5-ceRNA module Kaplan-Meier survival curves are shown in the top panel. The risk score distributions and individual plots of patient survival are shown on the middle and bottom panels. (**A**) The 5-ceRNA module could be used to divide the GBM patients in the training group into two different risk groups (log-rank test, *P* = 9.03 ×10^−3^). (**B**) The 5-ceRNA module could be used to divide GBM patients in the test group into two different risk groups (*P* = 0.02). (**C**) The 5-ceRNA module could be used to divide all of the 422 GBM patients into two different risk groups (*P* = 0.03). (**D**) The 5-ceRNA module also could be used to divide an independent set of GBM patients into two different risk groups (*P* = 0.02). The vertical gray lines represent the risk score thresholds in the survival analysis.

To identify synergistic, competing lncRNA modules, we used the Biclique algorithm. This algorithm was applied in a previous study of ceRNA networks [19] to identify ceRNA network modules within the LMCN. We found that MCM3AP-AS competed with three mRNAs (MATR3, CCHC14, and XPO1) and an lncRNA (MIR17HG) in a 5-ceRNA module (Figure 5B). These data were indicative of synergistic regulatory effects between the two lncRNAs. Within this module, XPO1 was associated with GBM prognosis [38]. To evaluate the association between the 5-ceRNA module and GBM survival, we integrated the expression profiles of the nodes into a risk model (Materials and Methods). Using the median risk score, we classified the training GBM patients into high- and low-risk groups (Figure 6A, *P* = 9.03 × 10^−3^). We then used the risk model to test the GBM patients using the same risk score threshold as that used to test the training patients. Similarly, the test group was divided into high- and low-risk groups (Figure 6B, *P* = 0.02). We used this 5-ceRNA module as a prognostic biomarker to divide all 422 GBM patients and obtained statistically significant results (Figure 6C, *P* = 0.03).

To evaluate whether the 5-ceRNAs module could be used as a prognostic biomarker, we applied the module to an independent GBM dataset (GSE7696). In this step, 76 patients with well-annotated clinical follow-up data were analyzed. We found that this module could also be used to divide the 76 GBM into high- and low-risk groups with significantly different survival times (Figure 6D, *P* = 0.02).

## DISCUSSION

MiRNAs are likely regulated by other RNAs that contain complementary miRNA binding sites such as lncRNAs and mRNAs. These miRNA sponges (ceRNAs) mutually regulate their expression via competing mechanisms, which are important for various tumor physiological and pathological processes. Typically, a ‘guilt by association’ strategy is employed to characterize lncRNA function [14, 15]. Analysis of gene expression data in combination with matched microRNA profiles indicated that the GBM ceRNA interaction network was associated with canonical oncogenic pathways [22]. However, this study was based upon a limited number of lncRNAs. Using a miRNA prediction method and co-expression analysis, another study identified lncRNA-associated ceRNA networks as well as prognostic lncRNAs across 12 cancers [19]. However, GBM samples were not analyzed in this study.

Several valuable ceRNA resources have been developed to facilitate functional analysis of lncRNAs such as starBase [39], DIANA-LncBase [40], LncACTdb [19] and miRSponge [15]. Here, we have used a more comprehensive approach to construct a functional lncRNA-mediated ceRNA network across 422 TCGA GBM samples. Our systematic analysis involved the integration of genome-wide lncRNA and mRNA expression profiles, miRNA-target interactions, functional analyses, and clinical survival analyses. We found that lncRNAs in the LMCN exhibited hub and bottleneck features, indicating they had regulatory associations with coding mRNAs across GBM pathology. MCM3AP-AS, which is involved in RNA processing and cell cycle-related functions, was significantly associated with survival. By exploring the highly competitive sub-network of the LMCN, we determined that MCM3AP-AS and MIR17HG comprised a ceRNA regulating module that competed with MATR3, ZCCHC14, and XPO1, which was associated with GBM prognosis [38]. By integrating the expression profile of this module into a risk model, we could stratify GBM patients from both the TCGA and the GSE7696 datasets into different risk groups. The results demonstrated that the cancer-associated LMCN can be used to accelerate biomarker discovery and GBM therapeutic development.

Blood-based biomarkers are critical for prediction of GBM patient survival. We observed MCM3AP-AS expression in white blood cells in a tissue-specific RNA analysis [41] and in several blood-associated RNA-Seq profiles (GSE33816, GSE64813, GSE64655, and GSE64831). These data indicated that MCM3AP-AS can be secreted into the circulation and is therefore a potential blood-based biomarker for GBM.

In summary, we have provided a comprehensive view of the LMCN across 422 TCGA GBM samples. The specific topological properties and synergistic, competitive effects of lncRNAs could reflect regulatory interactions with coding mRNAs in GBM.

## MATERIALS AND METHODS

### Expression profiles of lncRNA and mRNA in GBM

Genome-wide lncRNA and mRNA expression levels were derived from a recent study [42], which repurposed publicly available array-based data. Generally, exon array data were collected from the TCGA data portal [24]. The probe sets of the human exon array were reannotated to the human genome (version hg19). The expression values of lncRNA/mRNA were calculated by summarizing the background-corrected intensity of all of the probes annotated to this gene [24]. Quantile normalization was performed on the expression values for lncRNAs/mRNAs across patients. The ComBat method was used to remove potential batch effects [43]. Finally, we determined the expression of 8,132 lncRNAs and 17,364 mRNAs, and log2 transformed the values. An independent GBM expression dataset (GSE7696, Affymetrix Human Genome U133 Plus 2.0 Array) was used to validate prognostic biomarkers. The expression of a given lncRNA/mRNA gene was inferred from the mean expression of the different array probes. A total of 76 patients with well-annotated clinical follow-up information were analyzed in this step.

### Clinical characteristics of patients

The clinical and pathological data for the GBM patients were derived from the TCGA data portal. Detailed information for all of the GBM patients, the training dataset, and the test dataset are shown in Table 2. In total, 422 TCGA GBM patients with clinical follow-up information were retained in our framework. The clinical information of the GSE7696 dataset was derived from the Series Matrix File at the GEO database. The independent validation dataset consisted of 76 GBM patients.

### Identification of miRNA-target interactions

Interactions between miRNA-lncRNAs were identified using existing miRNA target prediction methods [26–28]. Putative miRNA-lncRNA interactions were identified using the miRanda algorithm (version November, 2010) [29] with the default parameters (Score ≥ 140 and Energy ≤ 7.0). The human mature miRNA sequences were downloaded from the miRBase (release 19) [44]. The lncRNA sequences were obtained from the GENCODE database (v19) [45]. We predicted miRNA target binding sites on the whole lncRNA sequences. The AGO-CLIP sequencing datasets [46] were used to distinguish the experimentally validated cases from the set of predicted miRNA-lncRNA interactions (Figure 1). Data for miRNA-mRNA interactions was downloaded from two highly reliable online miRNA reference databases, the TarBase (v6) [47] and the mirTarBase (release 4.5) [48]. Both databases store manually curated collections of experimentally supported miRNA targets.

### Identification of potential ceRNA interactions

To identify competing lncRNA-mRNA interactions, we used a hypergeometric test, which enabled evaluation of the significance of the shared miRNAs between each lncRNA and mRNA. The genome had a total number of N miRNAs, of which K and M were the numbers of miRNAs associated with the current lncRNA and mRNA, and x was the common miRNA number shared by the lncRNA and mRNA. The *P* value was calculated in order to evaluate the enrichment significance for that function as follows:

$$P=1-\sum_{t=0}^{x}\frac{\left(\begin{array}{c} K\\ t \end{array}\right)\left(\begin{array}{c} N-K\\ M-t \end{array}\right)}{\left(\begin{array}{c} N\\ M \end{array}\right)},$$ (1)

The *P* values were subjected to false discovery rate (FDR) correction. A FDR < 0.01 was used as the threshold.

### Co-expression analysis

To identify lncRNA-mRNA pairs, Pearson correlation coefficients were calculated based on the expression of the competing lncRNA-mRNAs pairs:

$$\rho_{X,Y}=\frac{\text{cov}(X,Y)}{\sigma_{X}\sigma_{Y}},$$ (2)

cov (*X*, *Y*) is the covariance of variables X and Y. *σ_X_* and *σ_Y_* are the standard deviations for X and Y, respectively. The threshold was set to a FDR < 0.01.

### Construction of the risk score model

To identify and evaluate the lncRNA signatures that could predict patient survival, patients were randomly assigned to either a training or test data set (Table 2). The sample sizes were the same in both groups, and there were no significant differences in clinical histological treatment (Chi-square test, *P* = 0.14) or history of neoadjuvant therapy (Chi-square test, *P* = 0.49). Univariate Cox regression analysis was used to evaluate the association between survival and candidate expression level. A risk score formula was the generated that integrated both the strength and positive/negative association between each candidate and survival. The risk score for each patient was calculated according to the linear combination of the expression values weighted by the regression coefficient from the univariate Cox regression analysis:

$$\text{RiskScore}=\sum_{i=1}^{n}r_{i}\text{Exp}(i),$$ (3)

in which *r_i_* was the Cox regression coefficient of candidate i from the training set, and n was the number of tested candidates. *Exp* (*i*) as the expression value of candidate i in a corresponding patient. The median risk score was used as a cut-off to divide patients in the the training dataset into high- and low-risk groups. Patients in the high-risk group were expected to have poor survival outcomes compared to patients in the low-risk group. This model and cut-off point was also applied to the test dataset in order to divide the patients into high- and low-risk groups.

### Network illustration and topological analysis

We used the Cytoscape software (v3.1.1) to construct and illustrate the ceRNA network. Several topological properties such as the node degree and the BC were analyzed using the built-in NetworkAnalyzer tool. The Maximal Biclique Enumeration algorithm was used to identify synergistic competing modules. This model consists of a complete bipartite graph in which an edge is realized from every vertex of a lncRNA set to every vertex of a mRNA set. Competing modules comprised of lncRNAs and mRNAs were identified using the Maximal Biclique Enumeration algorithm downloaded from the website of the Computational Biology Laboratory in the Department of Computer Science at Iowa State University.

### Analysis of BC

BC is a measure of the centrality of a node in a network. It is equal to the number of shortest paths from each node to all others that pass through the node. This property reflects the amount of control that a node exerts over the interactions of other nodes. The BC of a node can be given as: (number of shortest paths that pass through the node)/(the total number of all shortest paths in the network).

### Survival analysis

Kaplan-Meier survival analyses was performed for the two groups of patients, and statistical significance was assessed using log-rank tests (*P* < 0.05). All analyses were performed using the R 3.1.0 software.

## SUPPLEMENTARY FIGURE


