# Supplementary material for: Identification of prognostic biomarkers in glioblastoma using a long non-coding RNA-mediated, competitive endogenous RNA network
Source: Oncotarget. 2016 May 24;7(27):41737–47. doi: 10.18632/oncotarget.9569 (PMC5173092; doi:10.18632/oncotarget.9569)
Supplement: Supplementary file 1 [file oncotarget-07-41737-s001.pdf]

Identification of prognostic biomarkers in glioblastoma using a long non-coding RNA-mediated, competitive endogenous RNA network

Supplementary Materials

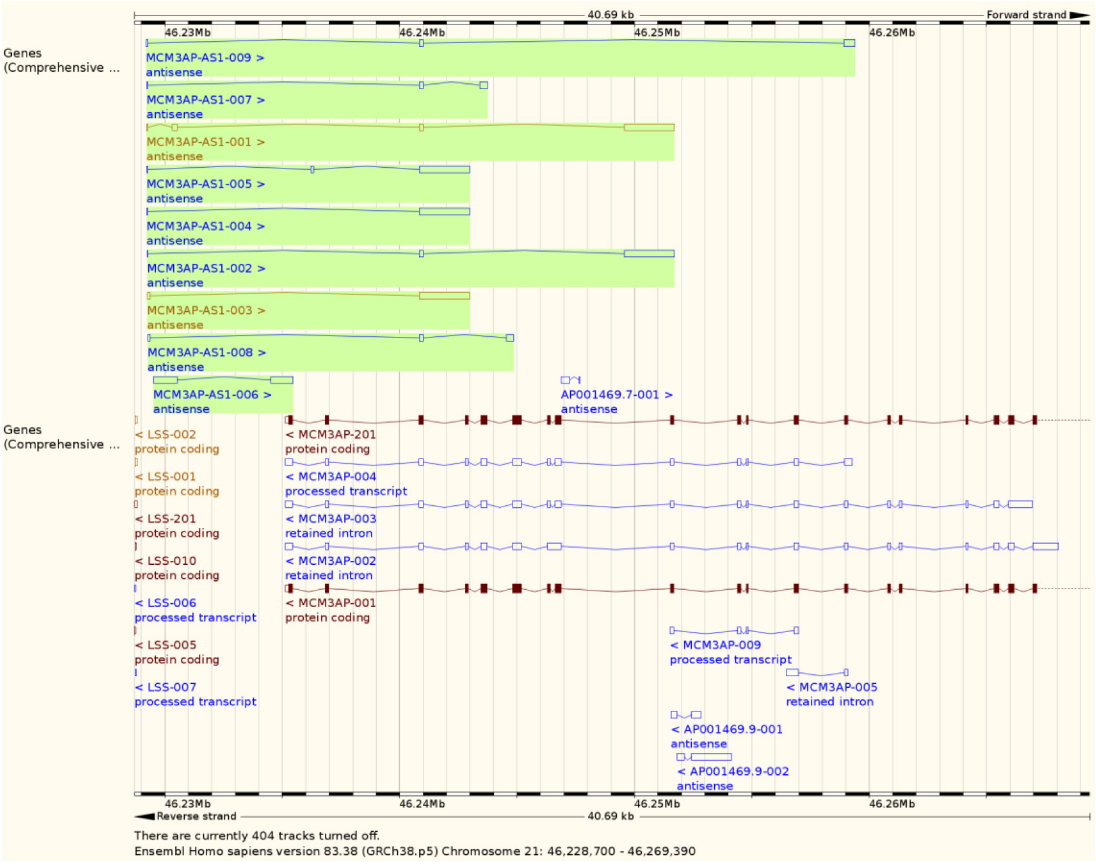

Supplementary Figure S1: Genomic location of MCM3AP and MCM3AP-AS according to Ensembl Genome Browser.
